# Multipolar traction pulley method combined with underwater endoscopic submucosal dissection for a large rectal laterally spreading tumor

**DOI:** 10.1055/a-2239-8558

**Published:** 2024-01-30

**Authors:** Fabien Pinard, Jérémie Jacques, Thomas Grainville, Martin Bordet, Louis Jean Masgnaux, Mathieu Pioche, Timothée Wallenhorst

**Affiliations:** 1 Gastroenterology and Endoscopy Unit, Cornouailles Hospital, Quimper, France; 2 Gastroenterology and Endoscopy Unit, Dupuytren University Hospital, Limoges, France; 3 Department of Endoscopy and Gastroenterology, Pontchaillou University Hospital, Rennes, France; 4 Gastroenterology and Endoscopy Unit, Edouard Herriot Hospital, Hospices Civils de Lyon, Lyon, France

Endoscopic submucosal dissection remains challenging, even with traction 1 to assist the procedure 2 . To overcome the decline in traction force as the dissection proceeds, an adaptive traction device, capable of being tightened to increase traction during the procedure, has shown interesting results 3 . Pulley methods have been described for early-stage gastric cancer 4 . However, since this first evaluation, no further study has been made in rectal and colonic locations.

We report the case of an endoscopic resection in a 73-year-old woman with a giant, rectal, granular mixed-type, laterally spreading tumor. We faced two difficulties. First, keeping good submucosal exposure without moving the patient, who was obese. Second, maintaining effective traction throughout the resection of this long lesion, which measured 10 cm from the oral to anal end. We decided to combine underwater dissection and multipolar traction with the pulley method.

As shown in Video 1 , after complete circumferential incision and trimming, we fixed a clip with a line attached and a rubber band to the anal side of the lesion ( Fig. 1 ). The rubber band was then fixed at both lateral sides of the lesion to obtain a multipolar traction effect. A line loop was passed over the original line, grasped with a clip, and fixed in the upstream colonic wall, beyond the oral edge of the lesion to maintain good traction during the entire procedure. Finally, we attached a surgical forceps to the line externally in order to apply constant weight. Dissection was performed with underwater saline immersion to counter unfavorable gravity effects. Pathology analysis revealed complete R0 resection of a 95 × 85 mm adenoma with intramucosal adenocarcinoma.

Multipolar traction pulley method combined with underwater endoscopic submucosal dissection for a large, rectal, laterally spreading tumor.Video 1

**Fig. 1:**
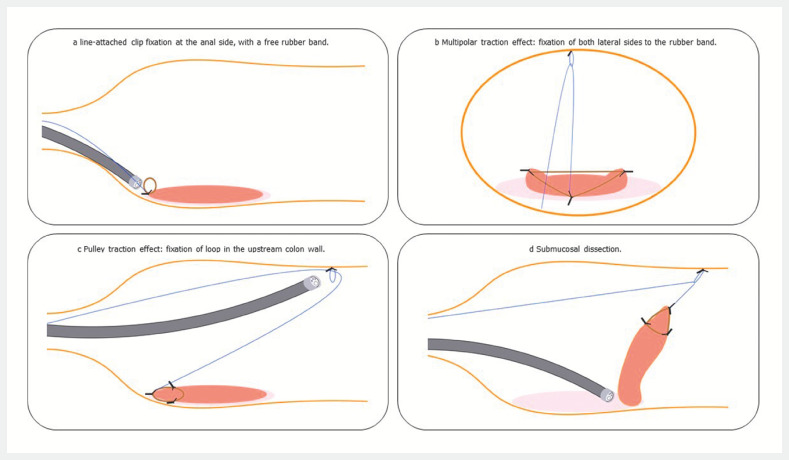
Schematic representation of submucosal dissection of a rectal laterally spreading tumor using multipolar traction with the pulley method. **a** Line-attached clip fixation at the anal side, with a free rubber band. **b** Multipolar traction effect: fixation of the rubber band at both lateral sides. **c** Pulley traction effect: fixation of a loop in the upstream colonic wall. **d** Submucosal dissection.

The multipolar traction pulley method combined with underwater resection could provide an additional traction tool to facilitate the endoscopic submucosal dissection procedure. Further studies are needed.

Endoscopy_UCTN_Code_TTT_1AQ_2AC

## Notes

Endoscopy E-Videos https://eref.thieme.de/e-videos
*E-Videos* is an open access online section of the journal *Endoscopy* , reporting on interesting cases and new techniques in gastroenterological endoscopy. All papers include a high-quality video and are published with a Creative Commons CC-BY license. Endoscopy E-Videos qualify for HINARI discounts and waivers and eligibility is automatically checked during the submission process. We grant 100% waivers to articles whose corresponding authors are based in Group A countries and 50% waivers to those who are based in Group B countries as classified by Research4Life (see: https://www.research4life.org/access/eligibility/ ). This section has its own submission website at https://mc.manuscriptcentral.com/e-videos .
